# Puerperal Laceration of the Cervix Uteri, and the Operation of Trachelorrhaphy as a Means of Cure*“This term was first employed (to my knowledge) by Dr. E. C. Dudley, now of Chicago, in a paper published in the *N Y. Medical Journal* for January, 1878, and is derived from *τράχηλοϛ*, neck, and *ραϕή* seam. I have adopted it because I think so important an operation should have a distinctive name of its own, and for convenience sake.”—Mundé, in *Am. Jour. Obstr.*, January, 1879.

**Published:** 1879-03

**Authors:** E. C. Dudley

**Affiliations:** Chicago, formerly House Surgeon to the Woman’s Hospital in the State of N. Y.


CHICAGO MEDICAL

JOURNAL & EXAMINER.

VOL. XXXVIII.—MARCH, 1879.—NO. 3.

[In these pages dimension and weight are expressed in terms of the Metric System, and temperature in degrees of the Centigrade Scale.]

### Original Communications.

### ARTICLE I.

### PUERPERAL LACERATION OF THE CERVIX UTERI, AND THE OPERATION OF TRACHELORRHAPHY * AS A MEANS OF CURE.

By E. C. DUDLEY, M.D., Chicago, formerly House Surgeon to the Woman's Hospital in the State of N. Y. Read before the Chicago Gynæcological Society, Jan. 31, 1879.

The two memoirs of Emmet † contain about all that is known of laceration of the cervix. Breisky,‡ of Prague, and Rokitansky, Jr.,§ of Vienna, have reported a number of successful cases. Breisky gives to Emmet the entire credit of having established the pathological significance and treatment of this lesion, but says that W. Roser,|| in 1861, described the same condition under the

* "This term was first employed (to my knowledge) by Dr. E. C. Dudley, now of Chicago, in a paper published in the *N. Y. Medical Journal* for January, 1878, and is derived from τράχηλος, neck, and 'ραφή, seam. I have adopted it because I think so important an operation should have a distinctive name of its own, and for convenience sake."—Mundé, in *Am. Jour. Obstr.*, January, 1879.

† *Am. Jour. of Obstetrics*, Nov , 1874. *Am. Practitioner*, Jan., 1877.

‡ *Wiener Med. Wochenschr.*, 1876.

§ *Wiener Medizinischer Presse*, July, 1876.

|| W. ROSER, *Archiv. für Heilkunde*, II., 1861.s.97.

name cicatricial ectropion of the cervix. The name cicatricial ectropion, formulated by Roser, involves a contradiction, for the presence of cicatricial tissue about the os could not cause ectropion or eversion, but by its tendency to contraction would have the opposite effect if any. Howitz,* of Copenhagen, with an experience of seventy-six cases, pronounces unqualifiedly for laceration as the essential cause of eversion, erosion, cystic degeneration and subinvolution, and for Emmet's operation as the only means of cure. A few other contributions in Germany† and America‡ have appeared merely as endorsements of the memoirs of Emmet. These have been designed not to be original, but to awaken the profession to the immense importance of the subject.

Either from absolute ignorance, or from profound prejudice, or from a carelessness which in the law would amount to malice, the most exhaustive gynæcological works in England,§ France|| and Germany¶ are absolutely silent. No published opposition, however, has appeared, perhaps because every man who has made investigation enough to warrant him in writing his experience, has been converted.

In the excellent paper of Mundé**, illustrated by twelve elegant and much needed lithographic plates, mention is made of Gardner's †† work on sterility, published in 1856, in which he finds that this author "repeatedly speaks of '*laceration of the cervix*' as productive of ulceration, hypertrophy of the cervix, endo-cervicitis and sterility," but points to no special means of diagnosis or operative treatment. This work contained two colored plates, in illustration of "fissure of the os," and "ulceration."

*History and Pathology.*—When we remember that the external os of the non-pregnant uterus has a diameter of about one-fifth of an inch, and that this diameter must be increased at term to

---

* Gynäcologiske og Obstetriciske Meddelelser, V.I., No. 3, 1878.

† Ruge and Veit Zeitschrift f. Geburtsh. u. Gynäkologie II., 1878.

‡ Wing, *Boston Med. and Surg. Jour.*, March, 1876. Baker, *Boston Med. and Surg. Jour.* Sept., 1877. Dudley, *N. Y. Med. Jour.*, Jan. 1878. Dudley. *Am. Jour. Ob.*, Oct., 1878. Harrison *Va. Med. Monthly*, Dec., 1874. Chase, Transactions of N. Y. S. Med. Society, Jan., 1878. Goodell, Transac. Med. Soc. State Pa., May, 1878. Mundé, *Am. Jour. Ob.*, Jan., 1879.

§ Barnes.

|| Lebold.

¶ Hegar, Kaltenbach, Schroeder.

** *Am. Jour. Obstetrics*, Jan., 1879.

†† The Causes and Treatment of Sterility, etc., by A. K. Gardner, A.M., M.D., N. Y., 1856.

the diameter of the child's head, we may readily suppose laceration of the cervix to be a common accident. It is now a recognized fact that some degree of laceration attends the majority of labors. This lesion is generally slight, and heals so readily that it is of little or no pathological significance. In lacerations through the anterior and posterior lips healing is the rule; this, Emmet says, is due to pressure of the lateral vaginal walls, which keep the "raw surfaces in close contact until they have firmly united."

Anterior laceration may extend so far as to invade the bladder, and make a vesico-utero-vaginal fistula, but even then healing often follows spontaneously, if attention be paid to cleanliness and position, leaving nothing to mark the laceration save a small vesico-vaginal fistula at the utero-vaginal attachment, or a utero-vesical fistula at the angle or bottom of the laceration, extending from this part of the cervical canal into the bladder. I have seen two cases of utero-vesical fistula due to this cause, in which healing had gone so far as to occlude the external os uteri. The menstrual blood in these cases passed away through the urethra.

Posterior laceration extending into the vaginal cul-de-sac may by creating cellulitis give rise to contracting cicatricial bands and effused lymph, and these may draw the uterus downward and backward, and fix it there in an almost incurable retroversion; permanent sterility in these cases is inevitable, and is doubtless due, in part at least, to obliteration of nature's reservoir for seminal fluid, the vaginal cul-de-sac.

*In lateral lacerations*, nature, instead of repairing the injury, resorts to a deception so artful that this lesion had been practically an unknown factor in uterine pathology until exposed by Emmet, in 1862. I refer to the substitution for the normal cervix of an apparently genuine * cervix, composed of everted intra-uterine and reflected vaginal tissue; a deception so complete that it sometimes fails to be detected even by the practiced eye. Indeed, if the differential diagnosis between normal and lacerated cervix were dependent solely upon ocular examination, cases would commonly arise in which increased size, congestion and a so-called "ulcer," would be the only differential signs. The formation of

* The false cervix here referred to includes only the vaginal portion of the entire cervix uteri.

this false cervix is indicated in the papers of Emmet, and what I shall add will only tend to corroborate his already established views.

Some of you will recall a paper by Dr. John Bartlett,* of Chicago, on the cervix before, during and after labor. The facts upon which Dr. Bartlett based his conclusions are entirely accessible, and are verified by the plates of Wilh. Braune,† of Leipzig, drawn from sections of the frozen gravid uterus. These conclusions which I introduce on the authority of Dr. Bartlett, harmonize with the history of lacerated cervix; they are, that from the moment of pregnancy the cervix as well as the body of the uterus enlarges to accommodate the growing fœtus, that the entire cervix from the first expands symmetrically with the body above, except a small portion which surrounds the external os; that this expansion very early in pregnancy obliterates the internal os, and converts the entire cervix into an inverted dome, which projects into the vagina, and whose walls are continuous with those of the body above. So that long before term a very large portion of the fœtal envelope is composed of evolved and expanded cervical tissue. Now, during labor there must be some point in this cervix above which all the tissues contract for the child's expulsion and below which they dilate for its exit. After delivery, examination shows, at a point above the normal site of the external os, about on a level with or a little below the plane of the utero-vaginal junction, the familiar, hard, unyielding ring vaguely called the os, regarded by Bartlett as the internal os, which seems impossible, since its location is at or below the vaginal junction, while that of the internal is considerably above. Neither can it be the external os, since its location is too high; it must, therefore, be between the two, at the very point just mentioned, above which contraction and below which dilatation occur in labor. It is, therefore, a temporary intra-cervical os, below which we must look for all that part of the cervix which during labor was compelled to undergo excessive dilatation, and expect there to find laceration, if it be present. Now, without great care, this portion of the cervix, which has been so stretched that it cannot

* *Chicago Med. Jour.*, Oct., 1873.

† Atlas of Topographical Anatomy, translated by Belamy, Philad., 1877.

immediately recover its contractile power, will be entirely overlooked, but it is always to be felt projecting into the vagina as a "flabby floating collar," not unlike "a section of large intestine," and as destitute of contractile power as the sphincter ani muscle after extreme forcible dilatation by the method of Van Buren; it is usually one or two inches in depth, and so deceptive to the touch that Dr. Bartlett, as he assures me, has often attempted to remove it by the finger for a clot of blood. After normal labor this slowly recovers its contractile power, and in a few days resumes its normal shape, and the external os is thereby restored.

But if bilateral laceration occur, nature has all the conditions for the formation of the false cervix already mentioned. The diverging flaps, as Emmet says, are at once forced in the directions offering least resistance, the posterior catching on the recto-vaginal wall and crowding backward into the vaginal cul-de-sac and the anterior forward in the axis of the vagina. The congested tissues about the temporary os, which we have called intra-cervical, meeting no resistance, now roll out, and this eversion, by obstruction to the circulation, causes the tissues to become so gorged with blood that they no longer have sufficient space for their accommodation within the uterus, and the eversion continues until tissue enough for the formation of a spurious cervix has been rolled out into the vagina, and until the temporary intra-cervical os has actually usurped the place of the normal os externum, which had been destroyed. When the patient assumes the upright position, all these distortions are exaggerated. Then the congested uterus by its weight settles lower in the pelvis and carries with it a reflected fold of the vaginal membrane, and the cervix is thus made to appear longer than it actually is. This deception may be exposed by placing the patient in the knee-chest position, when the uterus by its own weight will be carried towards the diaphragm, and the consequent unfolding of vaginal membrane will disclose the true utero-vaginal attachment, and not uncommonly expose a deep laceration on either side, which in the ordinary examination had been overlooked. The rolled out intra-uterine tissue of which the false cervix is composed, and the remains of the lacerated lips of the genuine cervix during involution, are so soft and easily molded that, resting in their prolapse upon the floor of the pelvis, they may gradually take the form of normal cervix, and the temporary intracervical os, which has usurped the place of the normal os externum, may appear at the very crown of this new cervix as a slit, perhaps not more than one-fourth inch long, the only remnant of a rent extending often an inch or more into the vaginal tissues on each side.

When the laceration is confined to one side, the deception is even greater, for, as shown by Emmet, the fundus in such cases is usually drawn towards the affected side by inflammatory contraction of the nearest broad ligament, the seat of a former cellulitis, which is a common result of laceration. The effect of this latero-version is to raise the uninjured side of the cervix a trifle higher in the pelvis and correspondingly to depress the injured side, thereby causing a reflection of the vaginal membrane on the depressed side, so that as in the bilateral injury the apparent os externum may seem to be in the very centre of the cervix when it is on the side, and to add to the confusion the sound entering at the side may, by passing to the horn of the opposite side of the uterus, appear to pass in the median line.

That laceration of the cervix causes marked outrolling of intra-uterine tissue, and a consequent permanent, passive congestion, is established beyond all question. The delicate intrauterine membrane is then in contact with the irritating acid secretion of the vagina, instead of the milder alkaline secretion of the uterus to which it is accustomed. But the mischief does not end here. The uterine supports soon prove unequal to the work of sustaining in position the uterus, now heavy from congestion, and it falls lower in the pelvis, where it remains generally in some form of version. The everted membrane, in contact with the posterior vaginal wall, is subject to the additional irritation of friction, an erosion or "ulceration" forms, and the mucous follicles, estimated by Tyler Smith * to number ten thousand in a normal virgin cervix, become diseased, and some of them pour out the familiar thick, colorless, viscid, ropy

* On Leucorrhœa, Am. Ed., page 38.

secretion, while others, their outlets having been occluded, become distended by their own secretion, and undergo cystic degeneration. I recall one case in which I must have emptied, by puncture with the Buttle's lance, hundreds of these little cysts in the treatment preparatory to operation. These cysts are generally present, and frequently in large numbers.

The uterine prolapse, in the words of Emmet, causes the broad ligaments to be "put upon the stretch," and by the consequent obstruction in the channels of blood and nerve supply which traverse these ligaments, we have additional cause of congestion and faulty innervation. Dr. Thomas * lays down the three following abnormal developments in the uterus as the basis of uterine pathology :

FIRST. *Disorders in innervation and circulation.*

SECOND. *Change in quantity of connective or muscular tissue.*

THIRD. *Change in position.*

He adds that either of these three being the primary lesion, the other two, singly or combined, may be its consequences. The first and third we already have ; the second is their inevitable result.

*Diagnosis and Treatment.*—Laceration of the cervix, until demonstrated by Emmet, was known only by its effects, and accordingly the following names were applied to designate their extent and character : "erosion," "excoriation," "granular erosion," "ulcer," "papillary erosion." These erosions, when exaggerated, were called cock's-comb granulations, or cauliflower growths, a condition simulating malignant disease, and when the cervical follicles were involved, follicular ulcer. In general "ulceration" became the popular and fashionable diagnosis. Even now, after seventeen years of demonstration to the contrary, the entire profession of Europe except a few Germans, and a large majority in America, fully asleep to the vast importance of Emmet's discovery, continue to mistake the cause for the effect, and treat the symptoms instead of the lesion itself. Never was a false theological dogma more thoroughly ground into a credulous people than this unfortunate though patent delusion of ulceration is now ground into the intel-

* Diseases of Women, page 208.

ligent medical profession. Except in specific disease, genuine ulceration is rarely if ever found on the cervix. Thomas* apologises for using the word, and says that "what is called ulceration on the cervix is called granular degeneration when it occurs under the lids."

When a case of uterine disease following parturition comes with a previous diagnosis of "ulceration or erosion with cervical enlargement and patulous os," we merely have to translate these words nto laceration of the cervix, and the diagnosis is correctly made. True, the "ulceration or erosion" is present, but that is a *fact*, not a *diagnosis*.

There is a form of erosion solely dependent upon an irritating discharge from the endometrium or vagina, apt to occur in feeble and badly nourished subjects, and not very uncommon in virgins; a condition analogous to the familiar erosion and excoriation produced by prolonged nasal discharges on the upper lips of children. Such an erosion is readily distinguished from that of laceration by the absence of eversion, absence of marked cervical enlargement, by the presence of a normally shaped os externum, and by physical examination soon to be described. It may be cured by removing the cause. It is generally associated with laceration, and when associated its treatment should be insisted upon as preparatory to the operation.

The presence of cystic degeneration of the mucous follicles of the external cervix, I believe to be pathognomic of laceration. These little cysts (already mentioned) feel to the touch like small shot scattered over the submucous tissues of the everted cervix. I have looked for them in hundreds of cases of uterine disease, but have never found one except on the lacerated cervix. My clinical experience also harmonizes with the histology of the intra-cervical membrane, as given by Stricker and Frey.

† "Lodged in the substance of these ridges (rugae cervicis), *excepting only in the lower and smoother part of the cervical canal*, we discover the so-called mucous follicles of the cervix."

‡ "Not unfrequently these little pits (mucous follicles) become occluded,

---

* Diseases of Women, page 303.

† Stricker's Manual of Histology, Am. ed., page 614.

‡ Frey's Histology and Histo-chemistry of Man, Am. ed., page 548.

and in consequence distended with mucus. They then present themselves in the form of small round vessels, known as the ovula nabothi."

* "These bodies (the ovula nabothi) are even found on the outer surface of the vaginal portion of the uterus."

If these follicles are normally found only within the cervical canal, it is clear that their presence in contact with the vaginal membrane must depend upon eversion, which I think never occurs to the extent of exposing the rugæ, except as the result of laceration, or of some other decided mechanical injury. Stricker finds the cysts or "ovula nabothi" "on the outer surface of the vaginal portion of the uterus," and concludes that they are distinct anatomical elements, although identical in structure, while Frey declares that the ovula nabothi are simply occluded mucous follicles, which Stricker says do not exist on "the outer surface." I therefore cannot escape the suspicion that the investigations of Stricker's "ovula nabothi" were made on the lacerated and everted cervix, not upon the virgin cervix.

The presence of the lesion may be detected in ordinary cases by the touch, but for the intelligent study of all cases, and for diagnosis in the more obscure, additional exploration is essential. The most reliable means are two uterine tenacula and Sims' speculum. The tenacula are absolutely essential, and I am fully convinced that their importance is not appreciated by many who, neglecting their use, not only fail in diagnosis, but question the accuracy of others who succeed. With these instruments, Emmet first untied the knot and revolutionized almost the entire pathology and treatment of cervical disease. The late Dr. Peaslee,† in his remarks on this subject before the N. Y. Co. Med. Soc., referring to the large number of cases of so-called ulceration, says "that they were not recognized, for none of us knew anything about them till Dr. Emmet told us. It was he who, in a happy moment, brought the anterior and posterior surfaces together with tenacula, and instantly demonstrated that what we all supposed an ulceration was nothing more or less than a laceration."

I quote the following from Dr. Emmet's first paper, published

* Stricker's Manual of Histology, Am. ed., pages 614 and 615.

† Mundé in American Jour. Obst., Jan., 1879.

in the *Am. J. Obst.*, Nov., 1874, twelve years after his first operation :

" November 27, 1862, I first operated for the relief of a double lateral laceration of the cervix, by freshening the surfaces and bringing together the anterior and posterior flaps with interrupted silver sutures. This patient had been an invalid for several years before coming under my care, and had been treated for menorrhagia and hypertrophy of the uterus, with an extensive erosion. She was undersize, of a naturally delicate constitution, and after a severe and protracted labor, with difficulty had given birth to a large child. Her general appearance indicated incipient phthisis, but no evidence of a tuberculous deposit could be detected. The uterus was some four inches in depth, and an erosion extended about two inches in diameter over an enormous cervix. With great care this erosion had been healed several times, by maintaining the recumbent position for a sufficient length of time, but a relapse to the former condition recurred in each instance shortly after beginning to exercise by walking. I had almost despaired of being able to offer her any permanent relief, and attributed my want of success to the condition of her general health. While making a digital examination one day I was puzzled to account for the greater width of the cervix in comparison to that of the body beyond, a condition I had for the first time appreciated. I placed her on the left side, and with Sims' speculum brought the cervix in view. I drew the posterior lips forward toward me with a tenaculum, but with no special purpose, when I was surprised to observe that it had decreased to nearly half its previous size. On lifting up the anterior lip with a tenaculum, in the other hand, so as to bring the two portions in approximation, the outline of a cervix presented, of nearly a normal size. The difficulty was at once apparent, for the parts had rolled back within the uterine canal, and a deep lateral fissure became evident, which extended on each side entirely through the cervix and beyond the vaginal junction. On separating the flaps and forcing them back to their former position, I saw the tissues gradually roll out, and the cervix again present its previous appearance. There could then be detected no appearance of laceration, and with the reduplication of vaginal tissue over the sides of the uterus, as I have already described, the cervix presented a normal length above its apparent junction with the vagina. The remedy at once suggested itself; the operation was performed with the aid of my assistant, Dr. G. S. Winston, and I believe Dr. T. G. Thomas was also present. On completing the operation, the uterus was five inches in depth, it rapidly reduced in size, and in time all evidence of local disease subsided, but she never entirely regained her general health. Some seven years after the operation, Dr. F. N. Otis, of this city, her family physician, detected a tuberculous deposit, and she has died of phthisis within a few months, having been ten years under my observation. For two years previous to her death she had resided abroad, but, as a friend, I was kept advised of her condition, and she continued free from uterine disease. I am fully satisfied, at the time of the operation her condition was so critical that it would have been but a question of a few weeks before a tuberculous deposit would have taken place. Although she never recovered fully the loss of vitality to which this injury had reduced her, yet her life was beyond question prolonged many years by the operation."

After the reading of this paper before the N. Y. Co. Med. Soc., Sept., 1874, Dr. J. Marion Sims said:

"When I went abroad in 1862, amongst the patients I turned over to the care of Dr. Emmet was the lady whose case forms the basis of the paper he has just read. She belonged to the upper walks of life, and had been under my charge for twelve or eighteen months. I remember the peculiarities of her case, so well described by Dr. Emmet, as vividly as if it were but yesterday. The bilateral laceration of the cervix, and the consequent eversion of the hypertrophied, congested cervical mucous membrane constituted at that time a difficult problem to solve. During the whole time that I observed this case no benefit resulted from local treatment, and I am sure that nothing short of the method so successfully adopted by Dr. Emmet could have been of the least service to her. I now only wonder that this operation had not been worked out sooner. When the perineum is lacerated, the necessity for its reconstitution is self-evident, and it is singular that the necessity for reconstituting the integrity of a lacerated cervix did not sooner force itself upon the surgeon. The operation as devised and practiced by Dr. Emmet is as simple, as safe and as certain in its results, as is the operation for a simple case of vesico-vaginal fistula. The same principles underlie each. The same free denudation of tissue, the same method of suture, the same after-treatment, and the same security from danger, belong to both alike.

"I have performed the operation often enough to speak in positive terms of its value. The discussion of the subject must, of necessity, be one-sided. There can be no objection, no opposition to the operation. We must accept it as Dr. Emmet has given it to us. We can't modify the operation; we can't change it; we can't improve it—for it is perfect—perfect in its method, and perfect in its results.

"We owe to Dr. Emmet a debt of gratitude for this valuable contribution to uterine surgery. Like all new operations it is likely to be abused, but the time will soon arrive when it will assume its place in the foremost rank of useful improvements.

"After the subject had been discussed by other members of the society, Dr. Marion Sims rose again, and said: 'I am personally so impressed with the importance of Dr. Emmet's paper in a practical point of view, and so pleased with the manner in which he has presented it to our consideration, that I beg leave to move a formal vote of thanks to Dr. Emmet for his most valuable contribution to surgery.'"

The preparatory treatment insisted upon by Emmet is absolutely essential to the best results, and has for its object the heal ing of the erosion by mild topical applications, the reduction of the congestion by the hot vaginal douche applied for twenty minutes twice daily, the puncture and emptying of the cysts, if any be present, replacement by mechanical support if indicated. The operation is strictly contra-indicated by the slightest evidence of peri-uterine cellulitis or pelvic peritonitis. If performed after suitable preparation it will be followed by prompt disappearance of congestion and rapid involution.

But is there no means of accomplishing these results without the application of sutures? Before proceeding, I desire, even at the risk of prolixity, to indicate once more the pivot upon which this cervical disease rests—contact of a congested intra-cervical membrane with the irritating acid secretion of the vagina, and its consequent exposure to friction and other mechanical injury. The disease then depends upon the hostile surroundings of an already congested membrane. Any reasonable plan of treatment must therefore look to replacment of the membrane to its normal position within the uterus, or to modifying it so that it may successfully resist its abnormal hostile surroundings. Much may be accomplished by the mild treatment already pointed out as preparatory to operation, and many of the milder cases may be cured. But a large number remains which can only be benefited, and still another large number upon which this sort of treatment has no effect whatever. Even those supposed to be cured are in danger of relapse. We have then, in this treatment, a valuable and indispensable aid to the operation, not a substitute. The everted membrane can neither be securely replaced by these means, or so modified as to endure its hostile surroundings. Again we ask, How shall this membrane be changed? There is but one reply, by cauterization, persistent and prolonged until the everted membrane has been replaced by a covering of dense, contracting, cicatricial tissue. If ordinary caustics are not powerful enough, we must invoke the caustic potassa and the actual cautery, and should these fail, amputation.

Ample authority of high order, for this practice, may be drawn from every capital in the civilized world. Dr. Thomas,* under granular ulceration, says:

"Should eversion of the cervix exist, the hæmorrhoidal mucous membrane should be at once removed by the scissors or by the curette, and should much

* Diseases of Women, page 358.

gaping of the os be present, the actual cautery or lunar caustic should be employed. I have spoken so freely elsewhere of the danger of producing cicatricial contraction of the canal and induration of the cervix that it requires no further mention here; in eversion however a certain amount of contraction is to be desired."

I am aware that this quotation does not fairly represent the present views of Dr. Thomas. He now recognizes the true cause of granular erosion, and, excepting Emmet, perhaps operates more frequently than any other man in the world.

Dr. Jacobi, of N. Y.,* recommends the actual cautery in cervical erosions, and claims to have been the first to use it in such cases.

Under "ulceration and enlargement," Dr. Byford† says:

"It is a very obstinate and discouraging state of the disease, but will usually yield to sufficiently energetic and long continued treatment. The boldness in the use of caustics necessary to the cure of such cases as these, requires strong nerves to institute and thoroughly execute."

Under "thoroughness and perseverance in the use of the caustic," he says:

"Failures occur very frequently on account of too little being done by the caustic. * * The treatment must be persevered in until the cure is complete."

Under the chronic or ultimate effects of the caustic is added:

"In very many cases where the application is made to the os uteri for several months that orifice becomes so small as to be of the size of a mere pinhole. * * We need not be embarrassed in our treatment by this occurrence, as we can easily dilate the os uteri to almost any extent by tents of compressed sponge or slippery elm bark bougies. By using one of these tents or bougies twenty-four hours before we desire to make the application, the opening will be large enough to answer all purposes."

These then are the agents to be substituted for Emmet's operation. But there is strong evidence going to show that the substitution of a hard-contracting scar for the cervical or endometrial membrane may irreparably incapacitate the uterus as a reproductive organ and exercise a very pernicious influence upon the general nervous system.

*It is axiomatic that the os uteri contracted to the size of a mere*

---

* *Medical Record*, March 9th, 1878.

† Byford on the Uterus, pages 99, 174, 180., 1871. [Since the reading of this paper Dr. Byford has informed me that he has almost entirely abandoned the use of nitrate of silver and topical applications to the uterus, and has substituted agents of a milder nature.—E. C. D.

*pin-hole is incapacited for the passage of the menstrual and seminal fluid.*

*It is evident that the attempt to enlarge a cicatricial canal by dilatation or incision will invariably be followed by a marked tendency, at least, to recontraction.*

*It is highly presumptive that a hard-contracting cicatricial cervix would not participate fully in the evolution of the gravid uterus, and that in labor, its rigidity would occasion delay and subject it to great danger of rupture.*

A priori, therefore, we may conclude that the cicatricial cervix is crippled, if not incapacitated for the natural performance of its menstrual and other reproductive functions.

But Dr. Emmet* asserts that cicatricial tissue on the cervix is frequently the cause of excessive nervousness and neuralgia in other parts of the body, and that, by the inclusion of nerve filaments, as in sensitive stump after amputation, it may serve as a constant and hidden source of nerve irritation. He has observed in his immense experience, covering thousands of carefully analyzed cases, that the anemic nervous neuralgic diathesis is peculiarly liable to be associated with cicatricial cervix. That the cicatrix and this diathesis have the relation of cause and effect I freely admit remains yet to be proved, and, to that purpose, I submit the following brief reports of two cases as evidence.

† A lady consulted one of the most distinguished ophthalmologists in America for a long-standing, severe and obstinate neuralgia of the eyeball. As the only possible means of relief extirpation of the eye was finally advised ; this operation the patient declined and the pain continued. She was subsequently operated upon by Dr. Emmet for laceration, and in the operation he removed a wedge-shaped piece of cicatricial tissue from the angle of the laceration which nature, in the vain attempt to bridge over the gap, had placed there. Immediate and permanent relief followed.

In April, 1878, I performed a similar operation upon a lady in La fayette, Ind. She had for years suffered from almost constant pain in the top of the head, and up to the time of the operation every resource of treatment had failed.

In this case the cervix was not eroded, but from the perseverance of some one in making caustic applications, it had suffered considerable loss of substance. The indurated tissue was very marked and abundant, so that I

---

* His two memors on laceration.

† I am not aware that this case has been published. This brief reference is drawn from the verbal report of Dr. Emmet. E. C. D.

was compelled to sacrifice an unusual amount of the cervix in its thorough removal. Entire relief from pain followed. A letter from her physician, Dr. E. D. Powers,* in the casual mention of the case, states that she now enjoys excellent health.

These cases are only two of a large number which strikingly illustrate Emmet's views of the effects of cicatrical cervix.

This subject of wholesale cauterization of the uterus is of vital importance and ought to be agitated until it is better understood, and therefore better appreciated. We certainly have facts and probabilities enough to suggest the question, whether the cautery which requires "strong nerves" on the part of the doctor, does not require stronger nerves on the part of the patient ?

In vivid contrast with the cautery is Emmet's operation, for it cures not by producing, but by removing the cicatrix.

During a residence of eighteen months at the Womans' Hospital in the State of New York, I had opportunity of observing and treating several hundred cases of uterine diseases. Of this number many came from their attending physicians with the diagnosis of hypertrophy and elongation of the cervix and had been sent in the expectation of amputation—an operation which, except for malignant diseases, is unknown in that institution, not having been performed, to my knowledge, for more than two years, in the combined services of Drs. Peaslee, Emmet, Thomas and Barker, although cases were continually presenting themselves in which the popular treatment would have been amputation. The brief report of a single case will illustrate them all.

An excellent description of the appearance presented by this case may be found in many of the popular gynæcological treatices under the head of Hypertrophy and Elongation of the Cervix.

It presented the apparent elongation of both anterior and posterior lips of the cervix,-which were actually protruding through the vulva. The utero-vaginal attachment appeared at about its normal distance from the vulva, so that the vaginal portion of the cervix seemed to point clearly to the diagnosis of extreme hypertrophy of the anterior and posterior lips. These lips were entirely separate from one another to the extent of their elongation, which appeared to be about three inches. In examining this patient for admission to the hospital, my first impression and also that of my senior assistant, Dr. VanVorst, was that of two large uterine fibroids protruding from the uterus into the vagina, but upon closer examination we agreed that it

*.I regret that the time is now too short to obtain a more definite history respecting the continued absence of pain.

was a case of true classical hypertrophy of the anterior and posterior lips. Knowing that Dr. Emmet had denied the possibility of such hypertrophy, we assigned the patient to his service, and waited his next visit. He promptly placed the patient in the knee-chest position, and introduced Sims' speculum; the vagina filled with air, the uterus was carried by gravity toward the diaphragm, the true utero-vaginal attachment now appeared in its true relation, about an inch from the end of the cervix, a deep laceration extending into the vaginal walls more than two inches on each side was evident. The depth of the uterine canal, measured by the probe, was nearly six inches; it was a case of sub-involution and procedentia, depending upon extensive laceration, of eight years' standing. After four weeks of preparatory treatment the rent was closed with about twenty silver sutures. Union was by first intention. In another month the canal measured three inches in depth, and before this patient was discharged, senile atrophy of the uterus was actually commenced. In another similar case, in which the apparent elongation was symmetrical, not specially involving either lip, but the entire cervix, the laceration was unilateral.

Barnes, of London, on Diseases of Women,* presents three notable cuts, illustrating what he calls hypertrophy and elongation, a condition in which he advocates amputation. Should he ever make proper use of the knee-chest position, the Sims' speculum and the two uterine tenacula these plates will become valuable, for he would then use them to illustrate *laceration of the cervix with procedentia and subinvolution.*

It has never been claimed that Emmet's operation is a panacea. It is only a safe, reliable, efficient and practical means of removing such abnormal conditions of the uterus as eversion and its consequent erosion, or "ulceration," congestion, "patulous os," subinvolution and uterine catarrh. More than this has never been asked or expected of it. That it will with entire safety accomplish some of these results in every instance, and frequently all of them, is shown in the history of hundreds of cases.

The operation is as safe and simple as that for vesico-vaginal fistula and requires equal skill. The indication for its performance is eversion of the intra-cervical membrane. Dr. Paul F. Mundé,† in his recent and notable paper, makes a strong plea for the early performance of this operation, believing it to be the most prompt and efficient treatment, in all cases of sufficient ex-

* Barnes, Diseases of Women, pages, 120, 121, 122.

† Am. Jour. Obstetrics. Jan. 1879.

tent to produce pathological effects. From his paper, I make the following quotations.

"At a meeting of the New York Obstetrical Society, held Nov. 20th, 1877, Dr. Alex. J. C. Skene, the President, expressed the opinion that there were many abnormal conditions of the cervix uteri, other than laceration, which could be cured better by this plastic operation than by any other means. While he did not explain himself more precisely, I drew the inference that he alluded to conditions very similar to those which I have been describing *(slight laceration)*, and which I have looked upon as, under proper conditions, calling for the operation.

"Another reason why trifling ectropia should not be allowed to go on for years unheeded and untreated, was recently advanced by Prof. Breisky,* of Prague, who has observed and operated upon four cases of laceration in which one of the everted lips had become carcinomatous in consequence of the irritation to which the exposed cervical mucosa was subjected. These observations are confirmed by Veit,† who, out of nine cases of carcinoma-cervicis, found three in which the disease originated in the enlarged glandular elements."

Emmet, in his memoirs, preceded Breisky in suggesting this relation of laceration to malignant disease.

Dr. Skene, in the Proceedings of the Medical Society of the County of Kings, June, 1878, advocates the substitution of silk for wire sutures. With these he had operated eight times in his office and sent the patients home in the street cars, to return in a week or ten days for removal of sutures; in every instance union was good. These were cases of slight laceration. This plan of Dr. Skene certainly simplifies and shortens the operation, and, if in respect of safety and perfect union it prove practical, it must inevitably remove all objection to the operation in the milder cases.

I would not, however, risk the danger of this plan, even in mild cases, until its safety had been demonstrated by a large number of cases. It certainly is not applicable to lacerations extending past the vaginal attachment.

The operation, like all other surgical procedures, may be performed contrary to indications, or without suitable preparation, or with insufficient skill, and therefore to the detriment of the patient. Then the misguided practitioner should pay the penalty of his own fault; the operation should not be compelled to do so for him.

---

* *Wiener Med.-chir. Rundschau*, August, 1877.

† Gynecol. Sec. German Congress of Physicians, 1877.

